# Association between ICS use and risk of hyperglycemia in COPD patients: systematic review and meta-analysis

**DOI:** 10.1186/s12931-021-01789-7

**Published:** 2021-07-08

**Authors:** Xiaofeng Pu, Liang Liu, Bimin Feng, Zhengji Zhang, Guojun Wang

**Affiliations:** 1 Department of Pharmacy, The Affiliated Hospital of Southwest Medical University, 25 Taiping Street, Luzhou, 646000 Sichuan China; 2 Department of Clinical Pharmacy, School of Pharmacy, Southwest Medical University, Luzhou, 646000 China

**Keywords:** COPD, Inhaled corticosteroids, Randomised controlled trials, Meta-analysis

## Abstract

**Background:**

The effect of inhaled corticosteroids (ICS) on risk of hyperglycemia in patients with chronic obstructive pulmonary disease (COPD) remains ambiguous. The aim of this study is to evaluate the association between ICS use and the incidence of hyperglycemia related adverse effects in COPD patients.

**Methods:**

Medline/PubMed, Embase, the Cochrane Central Register of Controlled Trials (CENTRAL), and ClinicalTrials.gov were searched from inception to 25 May 2020. Randomized controlled trials (RCTs) of ICS versus control (non-ICS) treatment for COPD patients reporting on risk of hyperglycemia were included. The Mantel–Haenszel method with fixed-effects modeling was used to calculate pooled relative risks (RRs) and 95% confidence intervals (CIs).

**Results:**

Seventeen RCTs with 43,430 subjects were included in the meta-analysis. Pooled results suggested that there was no statistically significant difference in the risk of hyperglycemia between the ICS group and the control group (RR 1.02, 95% CI 0.90–1.16, P = 0.76). In addition, no significant difference was noted in the effect on glucose level (RR 1.20, 95% CI 0.79–1.82, P = 0.40), risk of diabetes progression (RR 0.84, 95% CI 0.20–3.51, P = 0.81) and new onset diabetes mellitus (RR 1.0, 95% CI 0.88–1.15, P = 0.95) between the ICS group and the control group. These findings also were consistent across all subgroup analyses.

**Conclusions:**

Use of ICS does not have an effect on the blood glucose and is not associated with the risk of new onset diabetes mellitus and diabetes progression in patients with COPD. Further RCTs exploring the association between ICS use and risk of hyperglycemia in COPD patients are still needed to verify our results of this analysis.

**Supplementary Information:**

The online version contains supplementary material available at 10.1186/s12931-021-01789-7.

## Background

Chronic obstructive pulmonary disease (COPD) is a universal progressive inflammatory disease that is characterized by persistent respiratory symptoms and airflow limitation [1]. Exacerbations of COPD are important events in the course of the disease that often lead to an increased risk of death and have an impact on patients’ lung function and health status [2]. Long-acting bronchodilators, including β2 agonists (LABA) and muscarinic antagonists (LAMA), were recommended as the standard care to reduce the risk of severe exacerbations and to improve symptoms in patients with COPD [3]. In addition, Inhaled corticosteroids (ICS) treatment is suggested to only in Stage-D COPD with eosinophilia (Blood eosinophils > 300 cells/µl), according to GOLD 2020 guideline [4]. The place of ICS in the standard care of COPD is now much limited.

In the real-world environment, doctors frequently ignore clinical guidelines and the severity of the disease, and overusing ICS in COPD patients is common [5, 6]. Although the treatment containing ICS has a role in dual and triple therapy for COPD to reduce the risk of exacerbations and improve symptoms, ICS-related safety issues remain a serious concern. Treatment with ICS is associated with several adverse effects, such as pneumonia, fractures, and upper respiratory tract infection [7–9]. In addition, some observational studies revealed increased risk of onset and progression of diabetes, especially when higher ICS doses were utilized [10, 11]. However, some other studies did not indicate an increased risk of diabetes among users of ICS [12, 13]. Herein, there is currently no consensus on the association between ICS therapy and risk of hyperglycemia and diabetes.

A previous retrospective analysis evaluated whether there was an increased risk of new onset diabetes mellitus or hyperglycemia among patients with asthma or COPD treated with ICS in randomized controlled trials (RCTs), and found ICS therapy was not associated with increased risk of hyperglycemia or new onset diabetes mellitus [14]. However, the mean follow-up period of this analysis was only 217 days after the onset of ICS use and it published in 2012 [14]. Recently there are numbers of RCTs which investigate the treatment with ICS in COPD patients and report the adverse effects of rise in blood glucose levels, new onset diabetes mellitus and diabetes progression [15–19]. However, the incidence of these hyperglycemia related adverse effects are not consistent across the results. In addition, there is currently no meta-analysis to explore the association between ICS therapy and risk of hyperglycemia. Therefore, we perform this meta-analysis including all RCTs which record the hyperglycemia related adverse effects (rise in blood glucose levels, new onset diabetes mellitus and diabetes progression) among COPD patients with the ICS therapy to investigate whether treatment with ICS increase the risk of hyperglycemia in COPD patients.

## Methods

### Protocol and guidance

The study protocol was registered in the International Database of Prospectively Registered Systematic Reviews (PROSPERO; Registration No. CRD42020185288), and it was conducted in accordance with Preferred Reporting Items for Systematic Reviews and Meta-Analysis (PRISMA) statement [20]. An additional table file shows this in more detail [see Additional file 1: Table S1].

### Search strategy

We searched Medline/PubMed, Embase, the Cochrane Central Register of Controlled Trials (CENTRAL), from inception to 25 May 2020. We also searched ClinicalTrials.gov to identify ongoing or unpublished eligible trials. The geographic area and language were not restricted. Disagreements between two reviewers were resolved by discussion. The detailed search strategy was shown in an additional table file [see Additional file 2: Table S2].

### Inclusion criteria

The inclusion criteria were described below: (1) patients were diagnosed with COPD of any severity; (2) intervention was treatment containing ICS; (3) comparison was placebo or treatment containing non-ICS; (4) the data related to hyperglycemia were provided in the studies; (5) the type of study was randomised controlled trial (RCT).

### Exclusion criteria

We excluded the studies if they were case reports, or observational studies; if the patients complicated with allergic rhinitis, pulmonary infarction, pulmonary encephalopathy, bronchial asthma, pneumoconiosis, and active tuberculosis; if studies were published in reviews, abstracts, or protocols.

### Assessment of risk of bias in included studies

Two reviewers (XFP and LL) assessed the quality of each included study according to the Cochrane Handbook for Systematic Reviews of Interventions [21]. We assessed risk of bias according to the following items: random sequence generation, allocation concealment, blinding of participants and investigators, blinding of outcome assessment, incomplete outcome data, selective outcome reporting, etc. The risk of bias was assessed by two reviewers independently, and disagreements were resolved by discussion.

### Data extraction

Two independent reviewers (XFP and LL) utilized a standard data extraction form to extract data from the included RCTs. The studied data were pooled from the separate treatment arms when RCTs had more than two arms. The data were extracted from original articles and checked for accuracy by two reviewers.

### Data analysis

Herein, Stata 16.0 software was used to perform statistical analysis. We used risk ratios (RR) and their associated 95% confidence intervals (CIs) to assess outcomes, and considered a P value less than 0.05 to be statistically significant. Statistical heterogeneity was assessed using the I^2^ test, and the heterogeneity was considered significant when I^2^ ≥ 50%. A random effects model was used when significant heterogeneity was present; otherwise, a fixed effects model was utilized. The publication bias was assessed by visually inspecting the funnel plot and was detected via the Egger test, the Begg test, and the Harbord test.

### Subgroup analyses and sensitivity analyses

The subgroup analyses were performed to test the influence of daily dose, duration. When significant heterogeneity was observed in pooled effect estimates, the sensitivity analyses were used by removing one study at a time to explore whether the heterogeneity was significantly reduced.

## Results

### Eligible studies and study characteristics

**Fig. 1:**
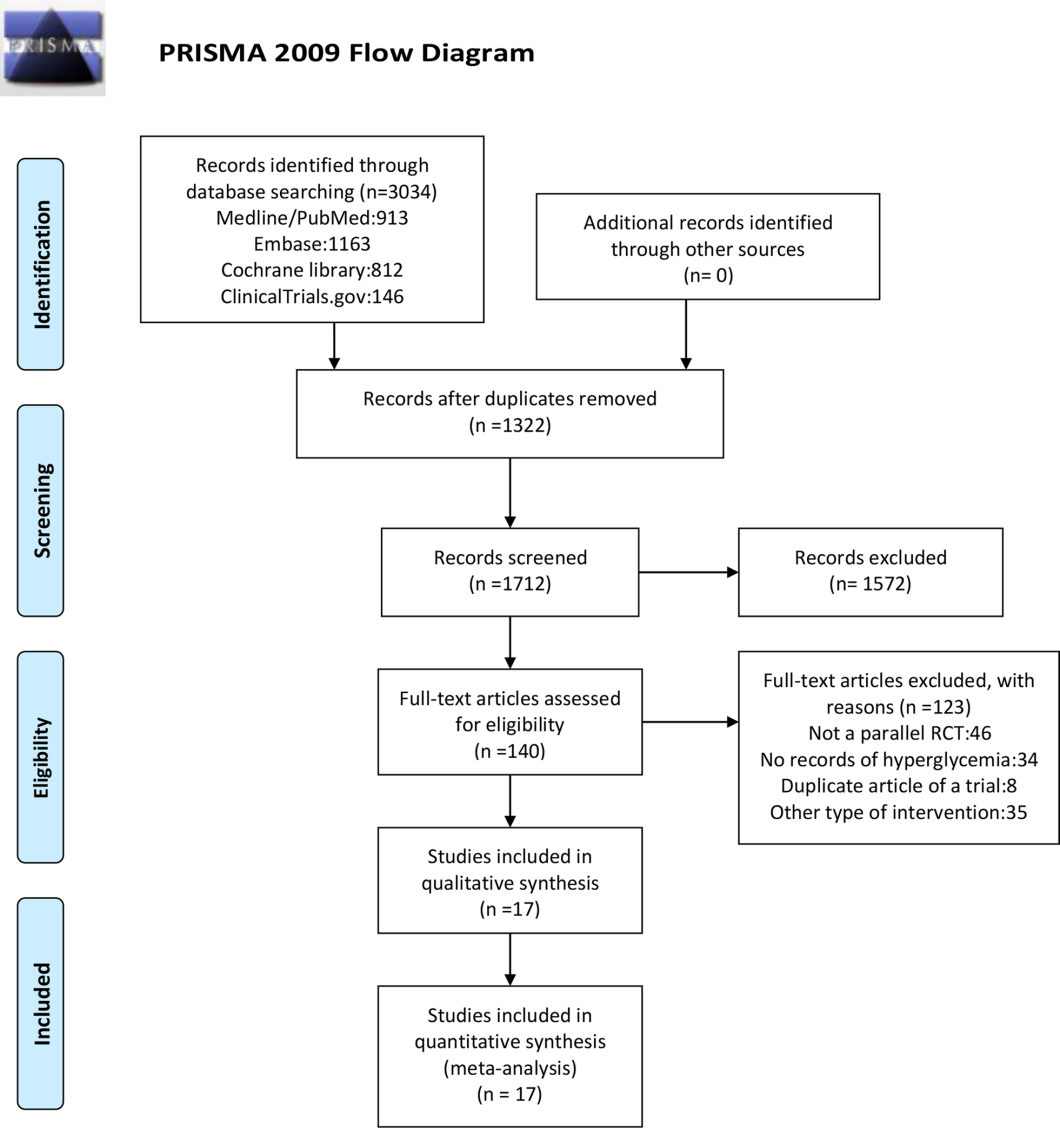
PRISMA flow diagram

Of the 3034 studies we retrieved from the aforementioned databases, we included 17 eligible trials [15–19, 22–33] in the final meta-analysis (Fig. 1). Detailed characteristics included in RCTs are presented in Table 1. The 17 included RCTs enrolled 43,430 subjects, of whom 26248received ICS treatment and 17,182 received non-ICS treatment. The RCT of Bhatt et al. [15] was included in both effect on glucose level subgroup and new onset diabetes mellitus subgroup, so the total number of subjects were 43,874 (26,389 with ICS treatment,17,485 with non-ICS treatment), as show in Fig. 3. The years of publication of these RCTs ranged from 2002 to 2018. Five studies used the low-dose ICS (fluticasone propionate < 250 μg/day; budesonide < 400 μg/day; beclomethasone dipropionate < 200 μg/day; fluticasone furoate < 100 ug/day) [34]. Eleven studies used the high-dose ICS (fluticasone propionate ≥ 500 μg/day; budesonide ≥ 800 μg/day; beclomethasone dipropionate ≥ 400 μg/day; fluticasone furoate ≥ 200 μg/day), and nine studies utilized medium-dose ICS.

**Table 1:** Characteristics of included studies

Study	Drug	Subjects, N	Male (%)	Mean age, years (SD)	Mean % predicted FEV1 (SD)	Treatment duration, weeks
Asai et al. (2015) [17]	SFC 50/250 µg, bid	26	25 (96)	64.7 (9.31)	1.983 (0.5797)	12
	Placebo	26	26 (100)	62.2 (8.06)	2.044 (0.4638)	
Zhong et al. (2015) [22]	QVA149 110/50 μg, qd	372	341 (91.7)	64.8 (7.8)	51.6 (12.8)	26
	SFC 50/500 μg, bid	369	331 (89.7)	65.3 (7.9)	52.0 (12.9)	
Bhatt et al. (2017) [15]	FF/VI 100/25 μg, qd	141	104 (77)	68.5 (8.0)	1.29 (0.43)	24
	VI 25 μg, qd	158	118 (77)	68.7 (7.7)	1.24 (0.42)	
	Placebo	145	119 (84)	68.2 (8.1)	1.30 (0.44)	
Dransfield et al. (2013) [16]	VI, 25 μg, qd	818	474 (58)	63·6 (9·4)	1·3 (0·5)	52
	FF/VI 50/25 µg, qd	820	476 (58)	63·6 (9·4)	1·3 (0·5)	
	FF/VI 100/25 μg, qd	806	453 (56)	63·8 (9·2)	1·3 (0·5)	
	FF/VI 200/25 µg, qd	811	467 (57)	63.7 (9.0)	1·3 (0·5)	
Kerwin et al. (2013) [23]	FF 100 μg, qd	206	132 (64)	62.7 (9.47)	46.9 (12.73)	24
	VI 25 μg, qd	205	140 (68)	63.4 (9.58)	49.9 (12.05)	
	FF/VI 50/25 μg, qd	206	135 (66)	62.8 (9.13)	48.4 (12.66)	
	FF/VI 100/25 μg, qd	206	137 (67)	62.3 (8.49)	47.8 (12.28)	
	Placebo	207	141 (68)	62.1 (8.80)	48.5 (12.46)	
Maltais et al. (2002) [24]	BUD 2 mg q6h	71	57 (80.2)	69.1 (8.7)	1.14 (0.45)	1.5
	Placebo	66	53 (80.3)	70.4 (8.9)	1.13 (0.44)	
Martinez et al. (2013) [25]	FF 100 μg, qd	204	150 (74)	61.8 (8.28)	48.4 (12.17)	24
	FF 200 μg, qd	203	151 (74)	61.8 (9.02)	47.1 (11.98)	
	VI 25 μg, qd	203	151 (74)	61.2 (8.62)	48.5 (12.89)	
	FF/VI 100/25 μg, qd	204	144 (71)	61.9 (8.79)	48.1 (12.85)	
	FF/VI 200/25 μg, qd	205	137 (67)	61.1 (8.58)	47.1 (12.76)	
	Placebo	205	152 (74)	61.9 (8.14)	48.3 (12.71)	
Sharafkhaneh et al. (2012) [26]	BUD/FM 320/9 μg bid	407	262 (64.4)	63.8 (9.4)	37.9 (11.8)	48
	BUD/FM 160/9 μg bid	408	264 (64.7)	62.8 (9.2)	37.6 (11.6)	
	FM 9 μg bid	403	229 (56.8)	62.5 (9.4)	37.5 (12.4)	
Siler et al. (2016) [27]	FF/VI 100/25 μg, qd	806	605 (75)	65.3 (8.58)	50.3 (10.33)	12
	VI 25 μg, qd	814	625 (77)	65.4 (9.02)	50.5 (10.33)	
Vogelmeier et al. (2013) [28]	QVA149 110/50 μg, qd	258	181 (70.2)	63·2 (8·2)	60·5 (10·5)	26
	SFC 50/500 μg, bid	264	189 (71.6)	63·4 (7·7)	60·0 (10·7)	
Vestbo et al. (2016) [29]	FF 100 μg, qd	4157	3053 (73.8)	65 (8)	59·6 (6·1)	96
	VI 25 μg, qd	4140	3053 (74.1)	65 (8)	59·7 (6·1)	
	FF/VI 100/25 μg, qd	4140	3112 (75.5)	65 (8)	59·7 (6·1)	
	Placebo	4131	3071 (74.7)	65 (8)	59·7 (6·1)	
Wedzicha et al. (2014) [30]	BDP/FOR 100/6 μg × 2, bid	601	408 (69)	64.6 (8.6)	41.9 (6.0)	48
	FOR 12 μg, bid	596	410 (69)	63.9 (8.6)	41.6 (6.0)	
Zheng et al. (2014) [31]	FF/VI 50/25 μg, qd	160	144 (90)	65.2 (8.41)	47.5 (14.21)	24
	FF/VI 100/25 μg, qd	161	149 (93)	65.1 (9.19)	49.6 (13.19)	
	FF/VI 200/25 μg,	160	145 (91)	62.7 (8.65)	48.2 (13.63)	
	Placebo	162	146 (90)	64.7 (8.78)	48.6 (13.39)	
Lipson et al. (2018) [18]	FF/UMEC/VI 100/62.5/25 μg, qd	4151	2766 (66.6)	65.3 (8.2)	45.7 (15.0)	52
	FF/VI 100/25 μg, qd	4134	2748 (66.5)	65.3 (8.3)	45.5 (14.8)	
	UMEC/VI 62.5/25 μg, qd	2070	1356 (65.5)	65.2 (8.3)	45.4 (14.7)	
NCT00857766 [32]	FSC 250/50 μg, bid	123	68 (55.3)	63.6 (8.92)		16
	Matching Placebo	126	74 (58.7)	63.5 (7.88)		
Wedzicha et al. (2016) [19]	QVA149 110/50 μg, qd	1678	1299 (77.3)	64.6 (7.9)	44.0 (9.5)	52
	SFC 50/500 μg, bid	1680	1258 (74.8)	64.5 (7.7)	44.1 (9.4)	
NCT03474081 [33]	FF/UMEC/VI 100/62.5/25 μg, qd	400	274 (68.5)	66.2 (8.08)		12
	Tiotropium 18 μg, qd	399	269 (67.3)	66.1 (7.78)		

*FF* fluticasone furoate; *VI* vilanterol; *SFC* salmeterol/fluticasone propionate; *QVA149* indacaterol/glycopyrronium; *BUD* budesonide; *FM* formoterol; *BDP/FOR* beclomethasone dipropionate/formoterol fumarate; *UMEC* umeclidinium; *FSC* Fluticasone Propionate/Salmeterol; *FEV1* forced expiratory volume in 1 second

### Risk of bias in included studies

**Fig. 2:**
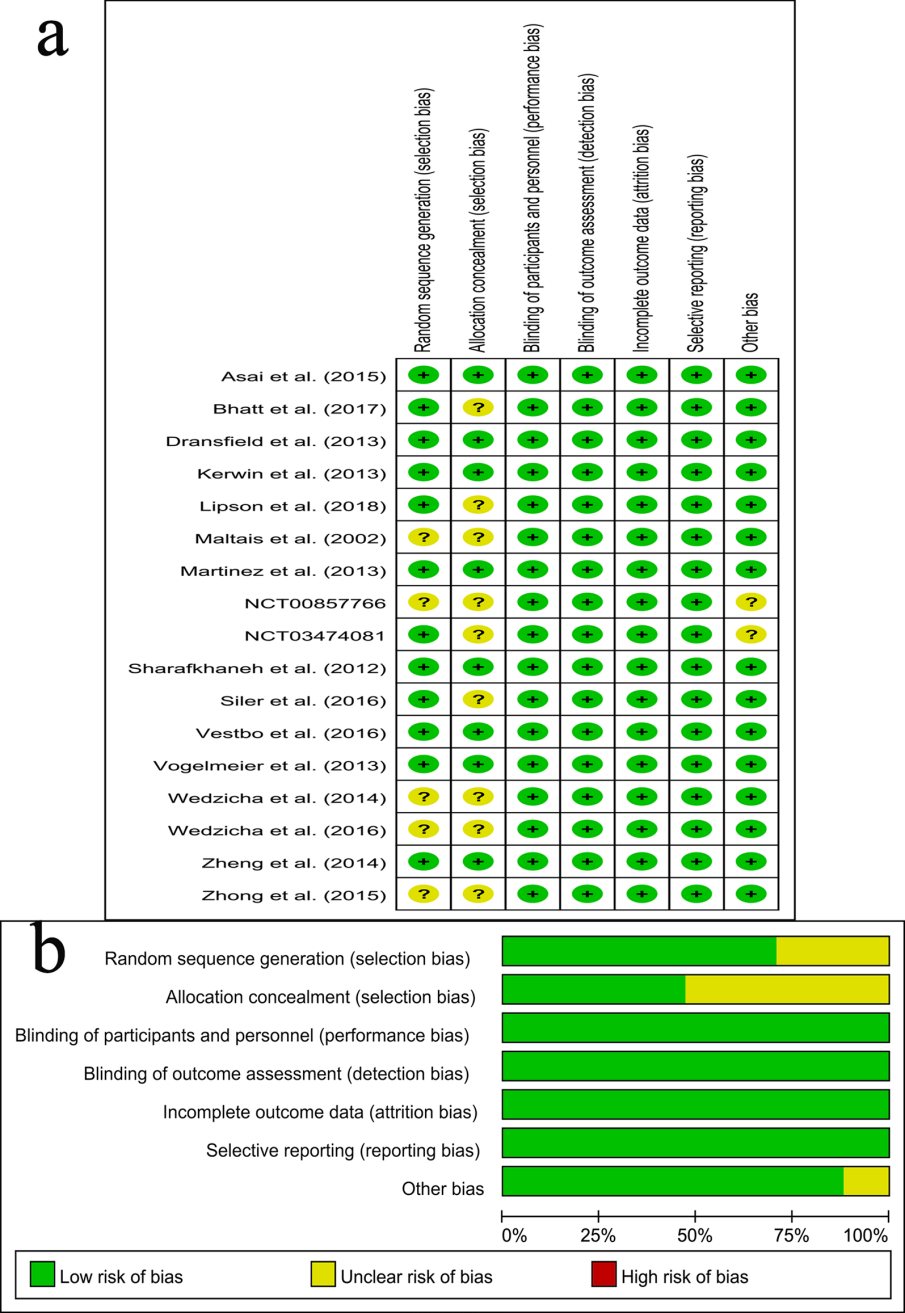
**a** Risk of bias summary for included studies, showing each risk of bias item for every included study. **b** Risk of bias graph presenting each risk of bias item as percentages across all included studies

Figure 2 showed the risk of bias. Eight trials had a low risk of bias. Five trials an unclear risk for random sequence generation. Nine trials had an unclear risk for allocation concealment, because it was not described in these trials. We did not find out other sources of bias in the fifteen trials, and they were unclear in the other two trials.

### Use of ICS and risk of hyperglycemia

**Fig. 3:**
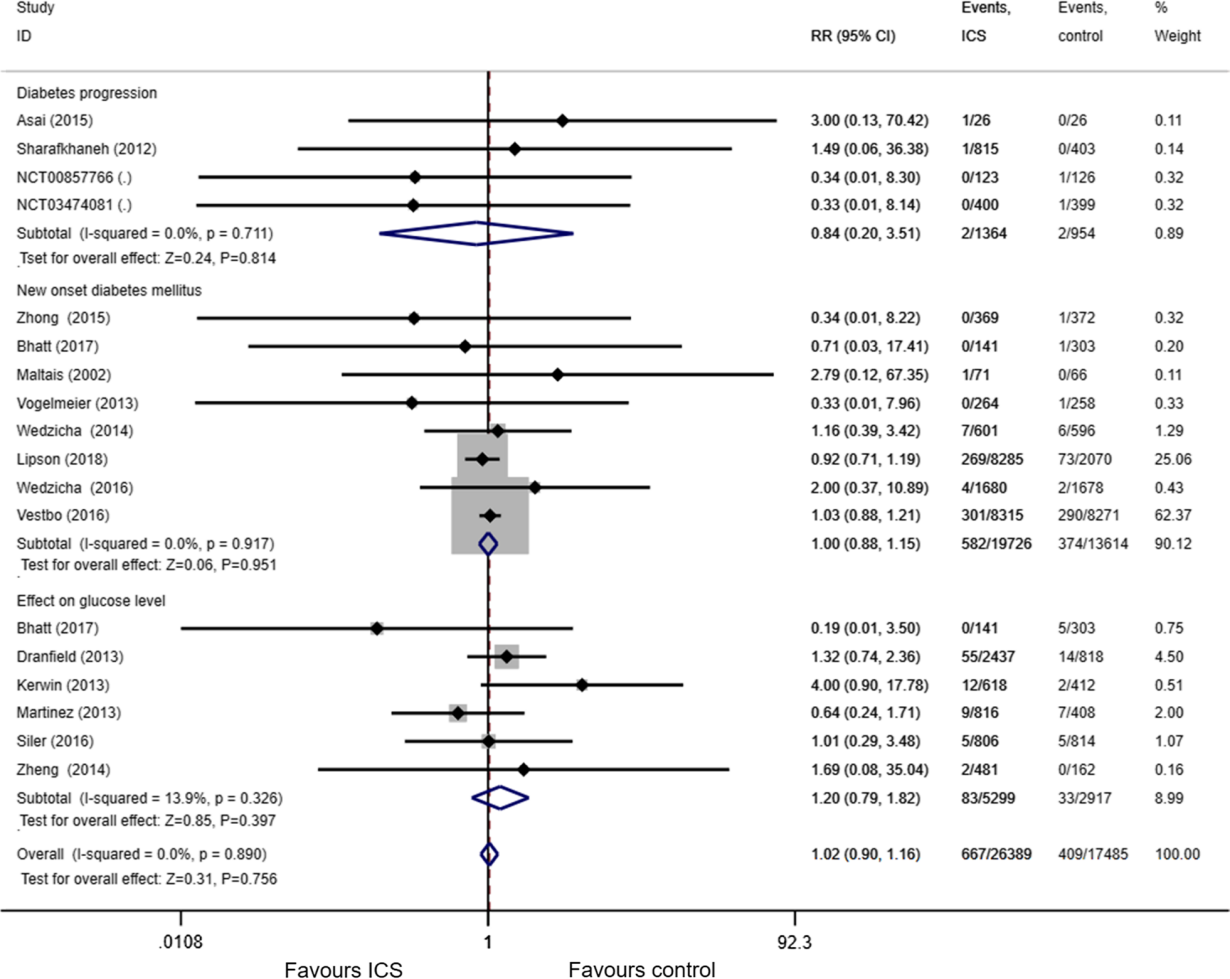
Forest plot for the effect of ICS on the risk of hyperglycemia. *RR* risk ratio; *ICS* Inhaled corticosteroids. %weight: The percentage of each study result in the overall result

**Fig. 4:**
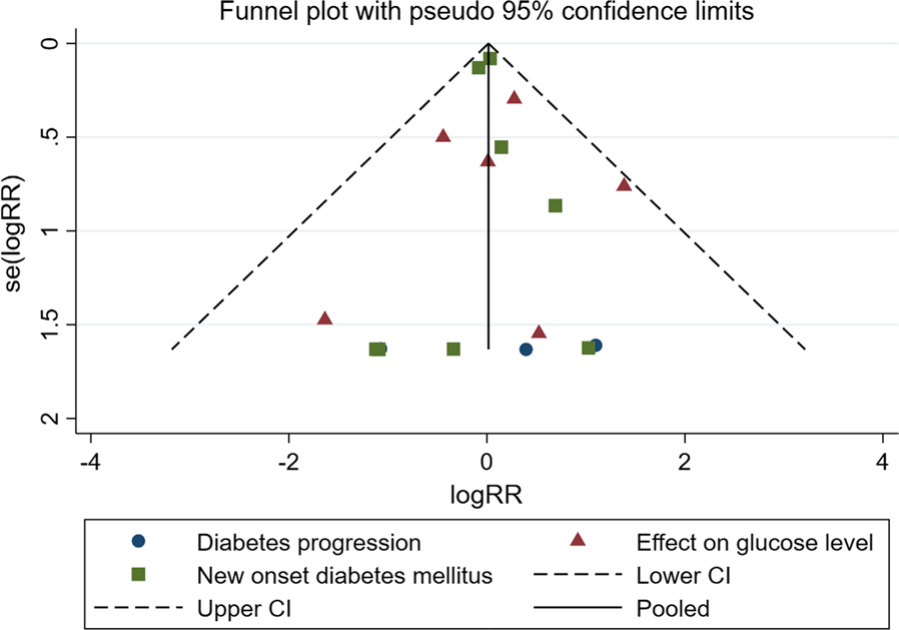
Funnel plot for the effect of ICS on the risk of hyperglycemia

All seventeen studies reported the adverse effects related to hyperglycemia. Among them, six studies reported the effect on glucose level, and four studies reported diabetes progression; new onset diabetes mellitus was recorded in eight studies. There was no statistically significant difference in the risk of hyperglycemia between the ICS group and the control group (RR 1.02, 95% CI 0.90–1.16, P = 0.76; Fig. 3). No statistical heterogeneity (I^2^ = 0%) was found in the pooled effect estimate. Funnel plot analysis showed no asymmetry (Fig. 4); additionally, the Egger test (P = 0.99), Begg test (P = 0.34), and Harbord test (P = 0.86) detected no significant publication bias.

Six studies reported the effect on glucose level. The pooled estimate revealed no significant difference between the ICS group and the control group in the effect on glucose level (RR 1.20, 95% CI 0.79–1.82, P = 0.40; Table 2). The results were consistent across all subgroup analyses, as shown in Table 2. The pooled RR for diabetes progression was 0.84 (95% CI 0.20–3.51, P = 0.81), and no statistical heterogeneity (I^2^ = 0%) was noted. Eight studies recorded the new onset diabetes mellitus. There was no significant difference between the ICS group and the control group in the risk of new onset diabetes mellitus (RR 1.0, 95% CI 0.88–1.15, P = 0.95). The results also were consistent across all subgroup analyses, as shown in Table 3.

**Table 2:** Subgroup analyses for the effect on the blood glucose

Subgroup title	No of trials	No of participants	Risk ratio (95% CI)	P Value	I^2^ (%)
Overall	6	8216	1.20 (0.79–1.82)	0.40	0
Daily dose					
Low dose	2	1949	1.20 (0.66–2.20)	0.53	22.3
Medium dose	2	4170	1.19 (0.72–1.95)	0.50	28.3
High dose	2	2097	1.38 (0.80–2.40)	0.25	0
Duration					
Less than 24 weeks	1	1620	1.01(0.29–3.48)	0.99	
At least 24 weeks	5	6596	1.22 (0.78–1.91)	0.37	30.3

**Table 3:** Subgroup analyses for new onset diabetes mellitus

Subgroup title	No of trials	No of participants	Risk ratio (95% CI)	P Value	I^2^ (%)
Overall	8	33,340	1.04 (0.88–1.15)	0.95	0
Daily dose					
Low dose	1	444	0.71 (0.03–17.41)	0.84	
Medium dose	2	26,941	1.00 (0.87–1.14)	0.99	0
High dose	5	5955	1.16 (0.53–2.54)	0.71	0
Duration					
Less than 1 year	4	1844	0.70 (0.16–2.98)	0.63	0
At least 1 year	4	31,496	1.01 (0.88–1.15)	0.91	0

## Discussion

### Main findings

This analysis showed that use of ICS did not significantly increase the risk of hyperglycemia, new onset diabetes mellitus and diabetes progression in patients with COPD. Moreover, these findings were consistent across all subgroup analyses. Dendukuri et al. [12] performed a cohort study to investigate the relationship between ICS and diabetes and their results did not indicate a significant increased risk of diabetes in COPD patients who used the ICS therapy. In a cross-sectional study, the influence of corticosteroid therapy in asthma on diabetes control was assessed, and this study revealed that ICS administered in low or mild doses do not affect fasting glycemia [35]. The insulin resistance is currently considered to be associated with not only type 2 diabetes (T2D) but also type 1 diabetes [36]. Borsi et al. conducted a quasi-experimental trial to investigate insulin resistance and the effect of ICS on insulin sensitivity in asthmatic patients, and the results of this trial indicated that there is no relationship between ICS and increased insulin resistance in asthmatic patients [37]. The findings of this meta-analysis are in line with the results of previous studies, mentioned above. The systemic bio-availability of ICS is considered to be minimal, so the metabolic complications involved in ICS use might be negligible [38]. This might explain our results for the no significant difference on the risk of hyperglycemia between ICS group and control group in COPD patients. However, there were some other studies found that use of ICS did increase the risk of hyperglycemia and diabetes. Saeed et al. [11] conducted a nationwide observational cohort study and demonstrated that ICS use was associated with a moderate increase (high ICS dose: HR 1.16, CI 1.01–1.32, p = 0.03) in the risk of T2D in COPD patients, but only for high-dose ICS use and BMI < 30 kg/m^2^. For the subgroup BMI ≥ 30 kg/m^2^, all exposure groups of ICS seemed to have a lower risk of T2D events. Another cohort study showed that long-term ICS therapy and high-dose ICS (mean daily dose ≥ 500 µg fluticasone propionate–equivalent) for COPD patients is associated with an increased risk of new onset diabetes and diabetes progression [10]. These studies demonstrated that there was association between high-dose ICS therapy and risk of diabetes and hyperglycemia, but moderate- and low-dose ICS groups showed no such association. Meanwhile, Dransfield et al. found that in patients with COPD, high-dose ICS therapy did not show superiority in reducing the acute exacerbations and improving the lung function compared to lower-dose ICS [16]. Our subgroup analysis results indicated the RR of the effect on glucose level in ICS vs. control increased from 1.20 to 1.38 with the ICS doses escalated. However, even for high-dose ICS group, the increase of the risk did not reach the level of statistical significance, compared to control group. Herein, use of ICS may not significantly increase the risk of new onset diabetes mellitus and diabetes progression, and not have an effect on the blood glucose. Further RCTs exploring the association between ICS use and risk of hyperglycemia are still needed to verify our results of this analysis.

### Strengths and limitations

This meta-analysis has some strengths. We followed the PRISMA statement and the recommendations of the Cochrane Collaboration to perform the study. In addition, we enrolled 17 RCTs with 43,430 subjects, so the combined results could be rigorous. However, our study also has several limitations. Firstly, the mean follow-up time of the included studies is around 30 weeks which is shorter than that of long-term observational studies, and this may result in no sufficient power to detect the observed excess risk. As Suissa et al. [39] reported that the incidence rate of diabetes was 14.2/1000/year in the ICS users with asthma or COPD, the insufficient follow-up period may lead to negative results due to lack of power. When new long-term followed RCTs complete and publish their results, we will combine them with the current results to draw a more rigorous conclusion. Secondly, the type of ICS used in the majority of the enrolled studies was fluticasone, so the subgroup analysis that investigated the effects of different types of ICS on the risk of hyperglycemia could not be performed. Thirdly, we did not make a distinction between the studies that used a placebo or studies that compared to LABA only use.

## Conclusions

Overall, use of ICS does not have an effect on the blood glucose and is not associated with the risk of new onset diabetes mellitus and diabetes progression in patients with COPD. Further RCTs exploring the association between ICS use and risk of hyperglycemia in COPD patients are still needed to verify our results of this analysis.

## Supplementary Information

**Additional file 1: Table S1.** The PRISMA checklist. 

**Additional file 2: Table S2.** The detailed search strategies for Pubmed and Embase.

## Data Availability

All data generated or analysed during this study are included in this published article [and its additional files].

## Abbreviations

COPD: Chronic obstructive pulmonary disease
ICS: Inhaled corticosteroids
LABA: Long-acting beta-agonist
LAMA: Long-acting muscarinic antagonist
RCT: Randomized controlled trial
CI: Confidence interval
PRISMA: Preferred Reporting Items for Systematic Reviews and Meta-Analysis
FEV1: Forced expiratory volume in 1 second
FF: Fluticasone furoate
VI: Vilanterol
SFC: Salmeterol/fluticasone propionate
BUD: Budesonide
FM: Formoterol
BDP/FOR: Beclomethasone dipropionate/formoterol fumarate
UMEC: Umeclidinium
FSC: Fluticasone propionate/salmeterol
