# Supplementary material for: Association between ICS use and risk of hyperglycemia in COPD patients: systematic review and meta-analysis
Source: Respir Res. 2021 Jul 8;22:201. doi: 10.1186/s12931-021-01789-7 (PMC8265114; doi:10.1186/s12931-021-01789-7)
Supplement: Supplementary file 2 — Additional file 2: Table S2. The detailed search strategies for Pubmed and Embase. [file 12931_2021_1789_MOESM2_ESM.docx]

**Search strategy for the PubMed**

 (((((((((((((COPD[Title/Abstract]) OR Chronic Obstructive Pulmonary Disease[Title/Abstract]) OR COAD[Title/Abstract]) OR Chronic Obstructive Airway Disease[Title/Abstract]) OR Chronic Obstructive Lung Disease[Title/Abstract]) OR Airflow Obstruction, Chronic[Title/Abstract]) OR Airflow Obstructions, Chronic[Title/Abstract]) OR Chronic Airflow Obstructions[Title/Abstract]) OR Chronic Airflow Obstruction[Title/Abstract]) OR "Pulmonary Disease, Chronic Obstructive"[Mesh])) AND ((((((((((((Budesonide[Title/Abstract]) OR Pulmicort[Title/Abstract]) OR mometasone[Title/Abstract]) OR flunisolide[Title/Abstract]) OR beclomethasone[Title/Abstract]) OR Foradil[Title/Abstract]) OR Advair[Title/Abstract]) OR Inhaled corticosteroid[Title/Abstract]) OR ICS[Title/Abstract]) OR fluticasone[Title/Abstract]) OR ciclesonide[Title/Abstract]) OR triamcinolone[Title/Abstract]))) AND ((((((randomized controlled trial[Publication Type]) OR controlled clinical trial[Publication Type]) OR randomized[Title/Abstract]) OR placebo[Title/Abstract]) OR randomly[Title/Abstract]) OR trial[Title/Abstract])

**Search strategy for the Embase**

#1 'chronic obstructive lung disease'/exp OR 'chronic airflow obstruction' OR 'chronic airway obstruction' OR 'chronic obstructive bronchitis' OR 'chronic obstructive bronchopulmonary disease' OR 'chronic obstructive lung disease' OR 'chronic obstructive lung disorder' OR 'chronic obstructive pulmonary disease' OR 'chronic obstructive pulmonary disorder' OR 'chronic obstructive respiratory disease' OR 'copd' OR 'lung chronic obstructive disease' OR 'lung disease, chronic obstructive' OR 'lung diseases, obstructive' OR 'obstructive lung disease' OR 'obstructive lung disease, chronic' OR 'obstructive pulmonary disease' OR 'obstructive respiratory disease' OR 'obstructive respiratory tract disease' OR 'pulmonary disease, chronic obstructive' OR 'pulmonary disorder, chronic obstructive'

#2 'inhaled corticosteroids':ab,ti OR 'inhaled glucocorticoid':ab,ti OR budesonide:ab,ti OR pulmicort:ab,ti OR 'mometasone furoate':ab,ti OR flunisolide:ab,ti OR beclomethasone:ab,ti OR foradil:ab,ti OR advair:ab,ti OR ics:ab,ti OR fluticasone:ab,ti OR ciclesonide:ab,ti OR triamcinolone:ab,ti

#3 'randomized controlled trial'/exp OR 'controlled trial, randomized' OR 'randomised controlled study' OR 'randomised controlled trial' OR 'randomized controlled study' OR 'randomized controlled trial' OR 'trial, randomized controlled'

#4 #1 AND #2 AND #3
